# Hospitals and the Dust Nuisance

**Published:** 1914-07-25

**Authors:** 


### HOSPITALS AND THE DUST NUISANCE.

At this season of the year it is appropriate to draw the attention of the public-health authorities in certain boroughs to the inadequate methods used in coping with street dust. It is unnecessary to do more than mention the connection between the blowing about of street refuse and the prevalence of sore throats and conjunctivitis. But this nuisance is one which also intimately concerns many hospitals in the Metropolis: we have in mind especially such hospitals as are situated on main thoroughfares, particularly in the north-eastern districts. We have noticed that there the problem seems more acute, though doubtless the authorities of other hospitals have cause for complaint. Take, for example, the City Road with the Moorfields Eye Hospital, the Lying-in Hospital, the Royal Chest Hospital, and the St. Mark's Hospital for Rectal Diseases; or the Kingsland Road, with the Metropolitan Hospital; or, again, the Hackney Road, with the Queen's Hospital for Children. All these institutions abut on the highway, and have no protection in the shape of front gardens. The traffic in these districts still consists of a large proportion of horse-drawn vehicles. The methods employed in watering the roads are inadequate for the purpose; we understand that reliance is still chiefly placed on the intermittent and sporadic attentions of water-carts. The waste of time and energy associated with these vehicles must be enormous, to say nothing of the expense entailed. Many of the side streets hardly ever get touched, and it cannot be a matter for surprise that the inhabitants and their children suffer from ill-health during the summer months. What is required is a much more thorough process of wetting the roads, and this can only be done by means of the hose-pipe. The provision of hydrants is probably already ample enough to allow of most streets being flushed, and the installation of a few more is no great engineering feat. The expense of this method would surely not be greater than that of the present horses and men employed for the carts. No doubt adequate flushing would mean that more water would be required, but this aspect ought also to be met successfully in view of the direct advantage which would ensue to the health and comfort of the community. Anyone who has experienced the conditions in the streets, say, of Hoxton, will bear witness to the marked difference between a well-watered street and a dry one on a hot summer's day.

To the hospitals themselves the dust nuisance is a serious matter; it militates against proper ventilation, and increases to a large extent the work of the staff. The harm dust may do to surgical and lung cases is so obvious that it need not be enlarged upon. The washing of the roads more thoroughly than is possible by means of a water-cart is no novelty. Theatre-goers returning home through the West End are familiar with the hosing process, which is employed nightly in certain streets. Even in those districts we have mentioned a certain amount of hose-work is done, but a far more extended use should be made of this mode of flushing the streets, and particularly on dry and gusty days. The secretaries of those institutions which are most affected by this nuisance should certainly bring this question to the notice of the borough surveyor and the medical officer of health. By persistent clamour something may eventually be achieved; for it cannot be denied that street flushing is a public-health measure of utility as great, if not greater, than many of the measures on which much public money is at present expended.

